# A systematic review of cannabidiol dosing in clinical populations

**DOI:** 10.1111/bcp.14038

**Published:** 2019-07-19

**Authors:** S.A. Millar, N.L. Stone, Z.D. Bellman, A.S. Yates, T.J. England, S.E. O'Sullivan

**Affiliations:** ^1^ Division of Medical Sciences and Graduate Entry Medicine, School of Medicine University of Nottingham, Royal Derby Hospital Derby UK; ^2^ Artelo Biosciences La Jolla CA USA

**Keywords:** cannabidiol, cannabinoid, dose, dosing, therapeutics

## Abstract

**Aims:**

Cannabidiol (CBD) is a cannabis‐derived medicinal product with potential application in a wide‐variety of contexts; however, its effective dose in different disease states remains unclear. This review aimed to investigate what doses have been applied in clinical populations, in order to understand the active range of CBD in a variety of medical contexts.

**Methods:**

Publications involving administration of CBD alone were collected by searching PubMed, EMBASE and ClinicalTrials.gov.

**Results:**

A total of 1038 articles were retrieved, of which 35 studies met inclusion criteria covering 13 medical contexts. Twenty‐three studies reported a significant improvement in primary outcomes (e.g. psychotic symptoms, anxiety, seizures), with doses ranging between <1 and 50 mg/kg/d. Plasma concentrations were not provided in any publication. CBD was reported as well tolerated and epilepsy was the most frequently studied medical condition, with all 11 studies demonstrating positive effects of CBD on reducing seizure frequency or severity (average 15 mg/kg/d within randomised controlled trials). There was no signal of positive activity of CBD in small randomised controlled trials (range *n* = 6–62) assessing diabetes, Crohn's disease, ocular hypertension, fatty liver disease or chronic pain. However, low doses (average 2.4 mg/kg/d) were used in these studies.

**Conclusion:**

This review highlights that CBD has a potential wide range of activity in several pathologies. Pharmacokinetic studies as well as conclusive phase III trials to elucidate effective plasma concentrations within medical contexts are severely lacking and highly encouraged.

**What is already known about this subject**

- Due to its favourable toxicity and side effect profile, cannabidiol is under increasing investigation in the commercial and medical industry to treat many clinical indications.

**What this study adds**

- This study identifies the wide active dosing range of cannabidiol (<1 to 50 mg/kg/d) within a variety of medical conditions including epilepsy, anxiety and graft‐*vs*‐host disease.
- This review indicates that studies that used higher doses tended to have better therapeutic outcomes compared to lower doses overall.
- This study identifies a strong existing need for dose‐ranging clinical studies to be conducted in which plasma concentrations can provide a better indication of the therapeutic range of cannabidiol.

## 1 INTRODUCTION

Cannabidiol (CBD) is a non‐intoxicating major constituent of the Cannabis sativa plant that has been increasing in interest due to its potentially diverse range of therapeutic properties and its favourable safety and tolerability profile.^1^ Side effects are generally mild and infrequent, such as sleepiness, diarrhoea or increased temperature. It is also reported that clinically significant drug‐interactions pose a low risk.^2^ There is no evidence for dependency or abuse potential with CBD use, as concluded by the World Health Organisation Expert Committee on Drug Dependence.^1^ The purported effects of CBD include analgesic, anti‐inflammatory, antioxidant, anxiolytic, anticonvulsant and cytotoxic effects, which are mediated through signalling mechanisms including the cannabinoid receptor 1 (weak agonist), the cannabinoid receptor 2 (inverse agonist), the serotonin 1a receptor (5‐HT_1A_), G protein‐coupled receptor 55 (GPR55), G protein‐coupled receptor 18 (GPR18) and the transient receptor potential cation channel subfamily V member 1 (TRPV1) receptors, amongst others.^3^

Clinically, CBD is being investigated in multiple disease states including neurodegeneration, anxiety disorder, orphan childhood diseases with a prevalence of <5 in 10 000 individuals (e.g. tuberous sclerosis complex) and addiction (ongoing trials in cannabis and cocaine craving).^4^, ^5^, ^6^ Epidiolex has recently become the first Food and Drug Administration‐approved CBD medicine, indicated for use in Lennox–Gastaut or Dravet syndrome (childhood epilepsy) by oral administration. Sativex is an oromucosal spray containing both CBD and δ‐9‐tetrahydrocannibinol, which is licenced in the EU and Canada for the treatment of multiple sclerosis associated spasticity. At the time of writing, there are 49 clinical trials registered on clinicaltrials.gov investigating CBD alone (either not yet recruiting, recruiting or active) and there have been at least a further 100 clinical trials previously registered containing CBD, indicating a significant clinical interest with an ongoing need to ensure that human volunteers engaged in these trials are given doses that are optimised for efficacy and safety. Surprisingly, none of the 49 currently registered trials have explicitly included a study design to investigate the dose‐ranging efficacy of CBD.

Hemp‐derived CBD is commercially available and is currently used as a health and food supplement commonly for anxiety and pain relief. This market represents a flourishing industry expected to rise financially and globally.^7^ However, the blurred lines between CBD as a licensed medicine and CBD as an over‐the‐counter remedy contribute to the overall lack of understanding of what dose of CBD may be considered *therapeutic*. This is further hampered by the lack of standardisation in over‐the‐counter CBD products and their unregulated labelled doses.

Despite the prevalence of CBD use and current hype, guidance on dose recommendations has not advanced and is not clear, additionally hampered by the striking lack of accessible pharmacokinetic and bioavailability data of CBD in humans.^8^ No published study to date has reported the absolute oral bioavailability of CBD in humans.^8^ Limited dose‐determination studies have left a paucity in data surrounding desired plasma concentrations to achieve minimum effective doses. Additionally, the lack of information on the role of different formulations and routes of administration on absorption are also apparent. The aim of this review was to comprehensively collate all published data relating to CBD administration in clinical populations to describe the range of CBD doses assessed across different pathological states.

## 2 METHODS

### 2.1 Search strategy

The systematic review was carried out in accordance with PRISMA (Preferred Reporting Items for Systematic Reviews and Meta‐Analyses) guidelines. A systematic search of PubMed, EMBASE (including MEDLINE) and clinicaltrials.gov was conducted to retrieve all articles reporting CBD administration in clinical populations using ‘CBD or Cannabidiol’ as search terms. Searches were restricted to ‘humans’ and ‘clinical trials and case reports’ in PubMed and EMBASE, with no restrictions on clinicaltrials.gov. The searches were carried out by 8 August 2018 by 2 independent researchers.

### 2.2 Eligibility criteria

The titles and abstracts of retrieved studies were examined by 2 independent researchers, and inappropriate articles were rejected. Inclusion criteria were as follows: an original, peer‐reviewed published paper that involved administration of CBD to a clinical population, or reported on clinicaltrials.gov, and included an outcome measurement to assess the efficacy of CBD i.e. improvement in disease. Exclusion criteria were: administration in healthy participants only; CBD administered in combination with other cannabinoids such as with δ‐9‐tetrahydrocannibinol or as whole cannabis extracts; article not in English; no stated concentration of CBD used; or no statistical results reported. The reference lists of included studies were hand‐searched for additional relevant studies.

### 2.3 Data acquisition and analysis

The included articles were analysed, and the following data extracted: sample size, clinical population/medical context; study design and length; administration route of CBD; source of CBD; dose of CBD; side effects; and primary outcome results. All data entry was checked by an additional independent researcher. Risk of bias of the 15 randomised controlled trials was assessed using the 2011 Cochrane Collaboration's tool for assessing risk of bias.

As this review included studies of participants of all ages (from infants to adults), dosing is reported in mg/kg of body weight to allow for comparison. Where not available as mg/kg (24 studies), dose was converted for adults using an average adult body weight of 62 kg.^9^ In only 1 publication, a case report on a child, an average child weight of 40 kg had to be used to convert reported mg/d dose into mg/kg/d.^10^

A positive effect of CBD was determined by the presence of a significant improvement in primary end points(s) or outcomes reported compared to placebo or baseline. A lack of positive effect was determined if no significant improvements were reported. Mixed findings were reported for example in case reports wherein some patients improved, others did not, or where a primary outcome was not specified (exploratory study) and in which some endpoints improved while others worsened (1 study) or remained unchanged.

## 3 RESULTS

**Figure 1:**
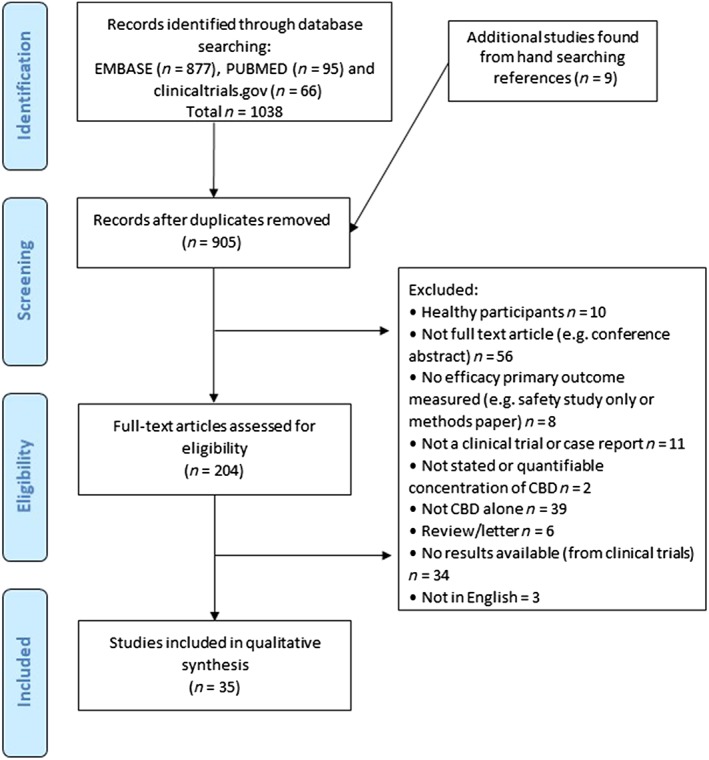
Flow chart of study retrieval and selection

**Figure 2:**
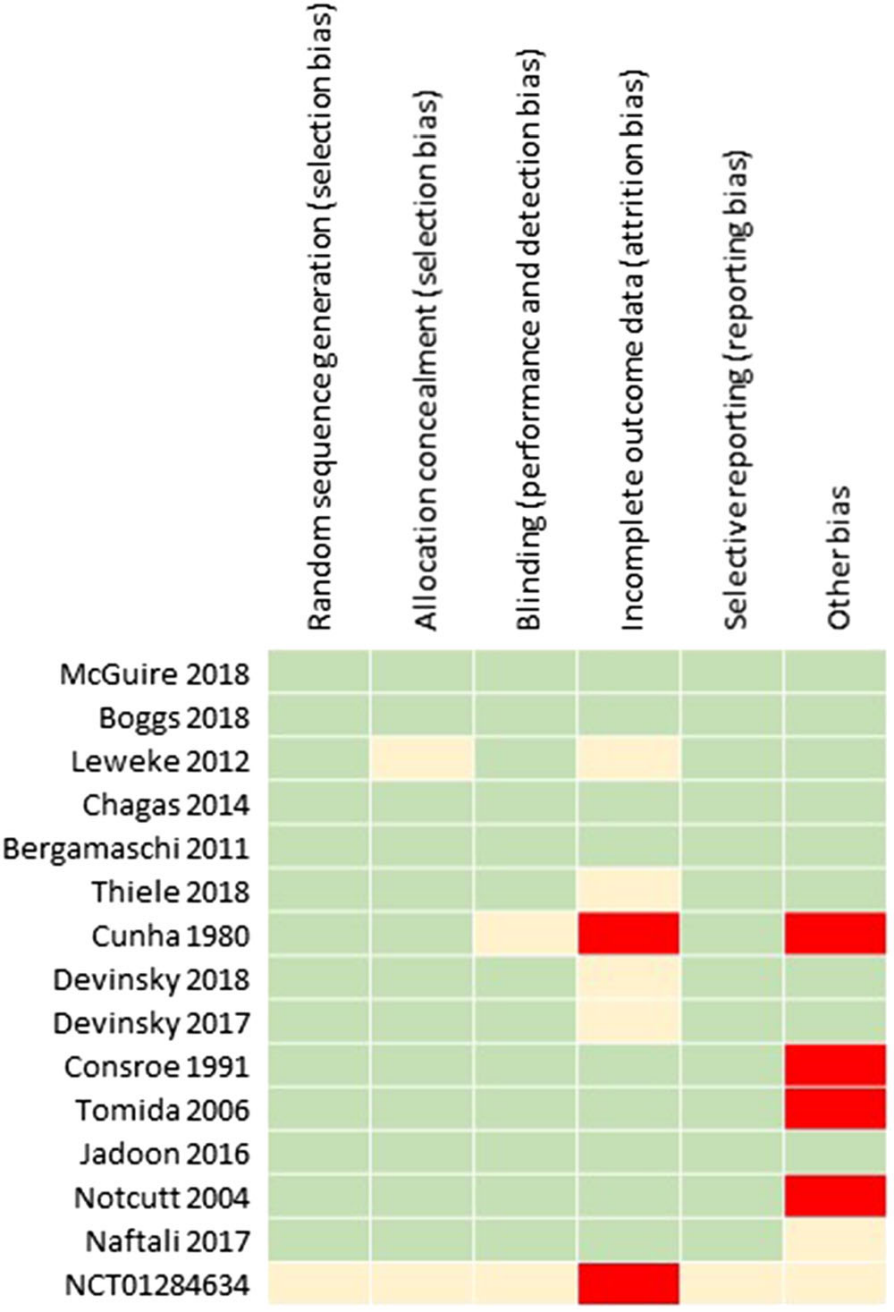
Risk of bias summary of the randomised controlled trials included in the systematic review. Green indicates low‐risk bias, red indicates high‐risk bias, and yellow indicates intermediate or unclear risk

The initial search yielded 1038 records, from which 896 abstracts were reviewed, and 35 articles were included in the final analysis, comprising a total number of 1223 participants. A flow chart of article retrieval and selection is presented in Figure 1. Fifteen studies were randomised controlled trials (RCTs), 8 were clinical trials but not both randomised and controlled in design (for example open‐label trials), and 12 articles were case reports/series. A description of each study is presented in tables 1, 2, 3 according to study design. Results of the risk of bias assessment of the RCTs are presented in Figure 2. A component of blinding was included in 74% of the RCTs . No study was reported with a high risk of selection bias, detection bias, or reporting bias. Overall, most information was from studies at low risk of bias. No study reported plasma concentrations of CBD. All studies reported oral administration of CBD, either as an oral solution (*n* = 11), capsules (*n* = 13), spray/sublingual (*n* = 4), or orally but unspecified (*n* = 6).

**Table 1:** Summary of included studies: randomised controlled trials

Study	Clinical population	Total n	Design	Trial length	CBD dose (mg) and approx. mg/kg/d^a^	Route of admin.	CBD source	Results^b^: Primary endpoint(s)	+ effect	Side effects
**McGuire, 2018 (NCT02006628)** 11	Schizophrenia, adults	88	Phase II exploratory double‐blind, parallel‐group, RCT. Add‐on therapy to anti‐psychotic drugs.	8 wk	1000 mg/d (16.7 mg/kg/d)	Oral solution	GW	Positive psychotic symptoms reduced. Negative, overall and general psychotic symptoms unchanged. Higher proportion of CBD treated patients rated as *improved*. No differences in functionality. No significant improvement in cognitive function except for motor speed. Overall reported as clinically significant improvements with CBD.	Yes	Rates of adverse events similar between CBD and placebo groups
**Thiele, 2018 (NCT02224690)** 12	Seizures (Lennox–Gastaut syndrome), ages 2–55 y	171	Double‐blind, phase III, RCT. Add‐on therapy to AEDs.	14 wk	20 mg/kg/d	Oral solution	GW	Monthly frequency of drop seizures decreased by a median of 43.9% in the CBD group, significantly more than in the placebo group	Yes	Diarrhoea, somnolence, pyrexia, decreased appetite, vomiting
**Devinsky, 2018 (NCT02224560)** 13	Lennox–Gastaut syndrome (epilepsy), ages 2–55 y	225	Phase III, double‐blind, RCT. Add‐on therapy to AEDs.	14 wk	10 or 20 mg/kg/d	Oral solution	GW	Significantly greater reduction in CBD groups in drop seizure frequency than in placebo	Yes	9% taking CBD had elevated liver aminotransferases. Somnolence, decreased appetite, diarrhoea, upper respiratory tract infection, pyrexia, vomiting.
**Boggs, 2018 (NCT00588731)** 14	Schizophrenia, adults	36	Double‐blind, parallel group, RCT. Add‐on therapy to anti‐psychotic drugs.	6 wk	600 mg/d (10 mg/kg/d)	Oral capsules	STI	No effect on cognition or symptoms	No	Similar rates between placebo and CBD, with exception of sedation which was higher in CBD group.
**Naftali, 2017 (NCT01037322)** 15	Crohn's disease, adults	19	RCT	8 wk	20 mg/d (0.3 mg/kg/d)	Orally, sublingual	On‐site	No difference in disease index	No	None observed
**Devinsky, 2017 (NCT02091375)** 16	Treatment resistant Dravet syndrome (epilepsy), aged 2–18 y	120	Double‐blind, RCT. Add‐on therapy to AEDs.	14 wk	20 mg/kg/d	Oral solution	GW	Reduction in frequency of convulsive seizures compared to baseline, significantly greater reduction than with placebo	Yes	Diarrhoea, vomiting, fatigue, pyrexia, somnolence, abnormal results on liver‐function: tests were higher in the CBD group than placebo
**Jadoon, 2016 (NCT01217112)** 17	Type 2 diabetes patients, adults	62	Double‐blind, RCT	13 wk	200 mg/d (3.3 mg/kg/d)	Oral	GW	No change in HDL‐cholesterol concentrations or glycaemic control.	No	Well tolerated
**Chagas, 2014** 18	Parkinson's disease, adults	21	Double‐blind exploratory RCT. Add‐on therapy to anti‐Parkinson's drugs.	6 wk	75 or 300 mg/d (1.25 or 5 mg/kg/d)	Oral capsules	THC	No effect on motor and general symptoms; 300‐mg dose improved well‐being and quality of life scores.	Mixed	None reported
**Leweke, 2012** 19	Schizophrenia, adults	42	Phase II, double‐blind, parallel‐group, RCT	4 wk	800 mg/d (max: 13.3 mg/kg/d)	NA	NA	Significant improvement of psychotic symptoms compared to baseline	Yes	Well tolerated
**Bergamaschi, 2011** 20	Generalised SAD, adults	24	Double‐blind, RCT	Acute	600 mg (10 mg/kg)	Oral capsule	STI and THC	Reduction in anxiety, cognitive impairment, discomfort in speech performance. Alert factors in anticipatory speech were also reduced.	Yes	None reported
**Tomida, 2006** 21	Ocular hypertension, adults	6	Double‐blind, 4‐way cross‐over, RCT	Acute	20 or 40 mg (0.3 or 0.7 mg/kg)	Oromucosal spray	GW	20 mg of CBD was ineffective, while 40 mg slightly increased intraocular pressure.	No	Mild—e.g. oral discomfort.
**Notcutt, 2004** 22	Chronic pain, adults	24	Double‐blind, 4‐way cross‐over, RCT. Add‐on therapy to pain medication.	8 wk	Approx. 9 sprays/d, equivalent of 22.5 mg/d (0.4 mg/kg/d)	Sublingual spray	GW	Symptom control or sleep duration was not improved with CBD; however, sleep quality was.	No	Mid—drowsiness, dry mouth
**Consroe, 1991** 23	Huntington's disease, adults	15	Double‐blind, cross‐over, RCT	6 wk	10 mg/kg/d	Oral capsules	US NIDA	CBD was ineffective	No	Similar between CBD and placebo
**Cunha, 1980** 24	Epilepsy, adults	15	Double‐blind, RCT study. Add‐on therapy to AEDs.	Up to 4.5 months	200–300 mg/d (5 mg/kg/d)	Oral capsules	NA	All but 1 patient improved condition	Yes	Well tolerated
* **NCT01284634** 25	Fatty liver disease, adults	25	Partially‐blinded, phase II, RCT	8 wk	200, 400 or 800 mg/d (3.3, 6.7, or 13.3 mg/kg/d)	Oral capsules	GW	No differences in liver triglyceride levels	No	Similar between CBD and placebo

a If not supplied, mg/kg/d was calculated based on average adult weight of 62 kg to enable comparisons.

b Significant compared to placebo/control (*P* < .05) unless stated otherwise.

* Registered clinical trial identifier: not published in any peer‐reviewed journal but results available from clinicaltrials.gov.

AEDs, anti‐epileptic drugs; CBD, cannabidiol; GW, GW Pharmaceuticals; HDL, high density lipoprotein; NA, not available; NIDA, National Institute on Drug Abuse; RCT, randomised controlled trial; SAD, social anxiety disorder; STI, STI Pharmaceuticals; THC, THC Pharm.

**Table 2:** Summary of included studies: clinical studies

Study	Clinical population	Total n	Design	Trial length	CBD dose (mg) and approx. mg/kg/d^a^	Route of admin.	CBD source	Results^b^: Primary endpoint(s)	+ effect	Side effects
**Rosenberg, 2017** 26	Epilepsy, 1–30 y	48	Open label clinical study	12 wk	2–5 mg/kg/d titrated up to 50 mg/kg/d or intolerance	Oral solution or by gastric tube	GW	Improvement in quality of life as well as some cognitive functions (memory and control)	Yes	Somnolence, drowsiness, fatigue
**Devinsky, 2016** 27	Drug‐resistant epilepsy, ages 1–30 y	137	Prospective, open‐label trial	12 wk	2–5 mg/kg/d, up‐titrated to 25 or 50 mg/kg/d	Oral solution or gastric tube	GW	Monthly motor seizures reduced by a median of 35.5% from baseline	Yes	Somnolence, fatigue, diarrhoea, decreased appetite, weight loss, status epilepticus (6%).
**Hess, 2016** 28	Drug‐resistant epilepsy in tuberous sclerosis complex, 2–31 y	18	Prospective study	6–12 months	5 mg/kg/d titrated up to 50 mg/kg/d if tolerated	Oral solution	GW	Decreased seizure frequency	Yes	Drowsiness, ataxia, diarrhoea
**Yeshurun, 2015 (NCT01385124)** 29	Cell transplant, (GVHD), adults	48	Prospective, phase II clinical trial	37‐day	300 mg/d (5 mg/kg/d)	Oral solution	STI	No patients developed acute GVHD. Significantly reduced risk ratio compared to historical case controls.	Yes	None reported
**Crippa, 2011** 5	Generalised SAD, adults	10	Double‐blind, placebo‐controlled study	Acute	400 mg (6.7 mg/kg)	Oral capsule	THC	Reduced subjective anxiety	Yes	None reported
**Hallak, 2010** 30	Schizophrenia, adults	28	Placebo‐controlled study	Acute	300 or 600 mg (5 or 10 mg/kg)	Oral capsules	Gift	No beneficial effects on selective attention	No	None reported
**Zuardi, 2009** 31	Psychosis in Parkinson's disease, adults	6	Open‐label pilot study	4 wk	150 mg/d, increased by 150 mg each week to a total of 400 mg/d (6.7 mg/kg/d)	Oral capsule	THC	Decrease in psychotic symptoms and Parkinson's disease rating compared to baseline	Yes	None reported
**Consroe, 1986** 32	Dystonic movement disorder, adults	5	Preliminary open pilot study	6 wk	100–600 mg/d, increased weekly (1.7–10 mg/kg/d)	Oral capsules	NA	Dose‐related improvement in dystonia disability	Yes	Mild—drop in standing blood pressure

a If not supplied, mg/kg/d was calculated based on average adult weight of 62 kg to enable comparisons.

b Significant compared to placebo/control (*P* < .05) unless stated otherwise.

CBD, cannabidiol; GW, GW Pharmaceuticals; GVHD, graft‐vs‐host disease; STI, STI Pharmaceuticals; SAD, social anxiety disorder; THC, THC Pharm.

**Table 3:** Summary of included studies: case studies

Study	Clinical population	Total n	Design	Trial length	CBD dose (mg) and approx. mg/kg/d^a^	Route of admin.	CBD source	Results^b^: Primary endpoint(s)	+ effect	Side effects
**Kaplan, 2017** 33	Refractory seizures in Sturge–Weber syndrome, children	5	Case‐series	14 wk	5–25 mg/kg/d	Oral solution	GW	Decreases in seizure frequency	Yes	Mild
**Warren, 2017** 34	Brain tumour related epilepsy, aged 17–40 y	3	Case series	2–10 mo	10–50 mg/kg/d	Oral	GW	Improvement in seizure frequency (n = 2) and severity (*n* = 3)	Yes	Diarrhoea
**Gofshteyn, 2017** 35	Febrile infection‐related epilepsy syndrome, children	7	Open‐label case series	Acute and up to 48 weeks	15–25 mg/kg/d	Oral solution	GW	Improvements in frequency and duration of seizures	Yes	Dizziness, decreased appetite, weight loss
**Shannon, 2016** 10	Anxiety and insomnia in PTSD, child	1	Case report	5 mo	25 mg/d (0.6 mg/kg/d)	Oral capsule and spray	CannaVest Corp	Increased sleep quality and duration, and decreased anxiety secondary to PTSD	Yes	None observed
**Saade, 2015** 36	Seizures, 10‐month old infant	1	Case report	6 mo	25 mg/kg/d	Oral solution	GW	Substantial reductions in seizures	Yes	None reported
**Chagas, 2014** 37	RBD in Parkinson's disease, adults	4	Case series	6 wk	75 mg/d (1.25 mg/kg/d)	NA	NA	Substantial reduction in RBD‐associated events compared to baseline	Yes	None reported
**Crippa, 2013** 38	Cannabis dependency, adult	1	Case report	10 d	300 mg/d increased to 600 mg/d (5–10 mg/kg/d)	Oral capsule	THC	Absence of withdrawal symptoms	Yes	None reported
**Zuardi, 2010** 39	Bipolar disorder, adults	2	Case series	30 d	600 mg/d increased to 1200 mg/d (20 mg/kg/d)	Oral	STI and THC	CBD was ineffective for manic episode	No	None observed
**Zuardi, 1995** 40	Schizophrenia, adult	1	Case report	4 wk	1500 mg/d (25 mg/kg/d)	Oral capsules	NA	Improvements in psychiatric ratings	Yes	Well tolerated; none reported
**Zuardi, 2006** 41	Treatment‐resistant schizophrenia, adults	3	Case series	30 d	40 mg/d, increased to 1280 mg/d (21.3 mg/kg/d)	Oral	GW	1 patient showed mild improvement to baseline and discontinuing treatment worsened symptoms	No	Well tolerated; none observed
**Snider, 1985** 42	Parkinson's disease, adult	1	Case report	4 wk	100–400 mg/d (3.3 mg/kg/d)	Oral	NA	Improvement of dyskinesia up to 200 mg/d, worsening of Parkinson disease symptoms with 300–400 mg/d	Mixed	Dizziness, drowsiness, increased Parkinson symptoms
**Snider, 1984** 43	Meige syndrome, adult	1	Case report	Long‐term	Initially 100 mg/d increased to 400 mg/d (6.6 mg/kg/d)	Oral	NA	50% improvement in spasm frequency and severity	Yes	Dry mouth, headache, sedation

a If not supplied, mg/kg/d was calculated based on average adult weight of 62 kg to enable comparisons.

b Significant compared to placebo/control (*P* < .05) unless stated otherwise.

CBD, cannabidiol; GW, GW Pharmaceuticals; PTSD, post‐traumatic stress disorder; RBD, rapid eye movement sleep behaviour disorder; STI, STI Pharmaceuticals; THC, THC Pharm.

Of the 15 RCTs, the range of doses investigated varied from <1 mg/kg up to 20 mg/kg per day (average 9 mg/kg/d).^11^, ^12^, ^13^, ^14^, ^15^, ^16^, ^17^, ^18^, ^19^, ^21^, ^22^, ^23^, ^24^, ^25^ Seven RCTs reported CBD efficacy (average dose 14 mg/kg/d),^11^, ^12^, ^13^, ^16^, ^19^, ^20^, ^24^ 7 studies describe neutral effects of CBD (average dose 5 mg/kg/d)^14^, ^15^, ^17^, ^21^, ^22^, ^23^, ^25^ and 1 study showed both positive and negative outcomes.^18^ In the remaining 8 clinical trials of various study design, 7 studies reported CBD positively (average dosing 23 mg/kg/d)^5^, ^26^, ^27^, ^28^, ^29^, ^31^, ^32^ and 1 study was neutral (8 mg/kg/d).^30^ Within the 12 case studies and case series, 9 described positive effects of CBD (average dosing 16 mg/kg/d),^10^, ^33^, ^34^, ^35^, ^36^, ^37^, ^38^, ^40^, ^43^ 2 were neutral (average dosing 21 mg/kg/d)^39^, ^41^ and 1 study described mixed results (3 mg/kg/d).^42^

Epilepsy was the most frequently studied medical condition, with all 11 studies describing beneficial effects of CBD in reducing the severity or frequency of seizures.^12^, ^13^, ^16^, ^24^, ^26^, ^27^, ^28^, ^33^, ^34^, ^35^, ^36^ Within the 4 conducted RCTs (*n* = 531), an average dosing of 15 mg/kg/d was used where CBD was administered successfully as an add‐on therapy to usual anti‐epileptic drugs.^12^, ^13^, ^16^, ^24^ Significant improvements were observed compared to placebo as an add‐on therapy. Within the other 3 clinical trials of prospective open‐label design (*n* = 203), CBD was administered at an average dosing of 42 mg/kg/d and significant improvements in quality of life and seizure frequency compared to baseline were observed.^26^, ^27^, ^28^ 3 case series and 1 case report (total *n* = 16) reported beneficial effects of CBD on seizure frequency, duration and severity with an average administered dose of 21 mg/kg/d.^33^, ^34^, ^35^, ^36^

Seven studies were conducted in the context of schizophrenia and bipolar disorder. Within the RCTs, 2 conducted with an average dosing of 15 mg/kg/d over 4 or 8 weeks reported positive reductions in psychotic or psychiatric symptoms and a better side effect profile (*n* = 130).^11^, ^19^ One of these compared CBD against an active control (amisulpride), and the other as an add‐on therapy to usual medication compared to placebo as an add‐on therapy. However, a third RCT employing CBD as an add‐on therapy did not report any improvements in cognition or symptoms of schizophrenia after a lower average dose of 10 mg/kg/d over 6 weeks (*n* = 36).^14^ An acute dose of 5 or 10 mg/kg/d did not improve selective attention in a placebo‐controlled trial of 28 schizophrenia patients.^30^ A number of case studies have also been conducted by Zuardi and colleagues in this medical context. In 2 patients with bipolar disease, 20 mg/kg/d was ineffective in treating manic episodes.^39^ CBD was similarly unable to improve symptoms in 3 schizophrenia patients, although 1 patient described mild improvement.^41^ Another case report described improvement in psychiatric ratings following an average dose of 25 mg/kg/d over 4 weeks.^40^

Results are mixed within Parkinson's disease studies. Within an RCT in 21 patients, 1.25 or 5 mg/kg/d CBD had no effect on motor and general symptoms. However, the 5 mg/kg/d dose improved well‐being and quality of life scores.^18^ The remaining studies are case studies in which CBD decreased psychotic symptoms and Parkinson's disease ratings (*n* = 6; 7 mg/kg/d),^31^ improved rapid eye movement sleep behaviour disorder (*n* = 4; 1 mg/kg/d),^37^ decreased dyskinesia with 2 to 3 mg/kg/d doses (*n* = 1), but exaggerated Parkinson's disease symptoms with 5 and 7 mg/kg/d doses.^42^

CBD did not change therapeutic outcome variables in a double‐blind RCT in Huntington disease patients compared to placebo (*n* = 15; 10 mg/kg/d for 6 weeks),^23^ but improved dystonia disability in an open pilot study (*n* = 5; 10 mg/kg/d for 6 weeks),^32^ and improved spasm frequency and severity in a case report in 1 patient with Meige syndrome (7 mg/kg/d).^43^

Within the RCTs, CBD did not significantly change the primary outcomes in diabetes (*n* = 62), Crohn's disease (*n* = 19), ocular hypertension (*n* = 6), chronic pain (mostly neuropathic; *n* = 24), or fatty liver disease (*n* = 25).^15^, ^17^, ^21^, ^22^, ^25^ However, an average dose of 2.4 mg/kg/d (range 0.3–13.3 mg/kg/d) was used in these studies, which is very low in the clinical and clinical trial setting compared to other studies. Low doses (10 mg/kg) did, however, produce positive responses in generalised social anxiety disorder (SAD) in a double‐blind RCT in 24 patients.^20^ Likewise, in another double‐blind placebo‐controlled study, a dose of 6.7 mg/kg reduced subjective anxiety in 10 adults with generalised SAD.^5^ Additionally, in a case report in a child, 0.6 mg/kg/d increased sleep quality and duration, and decreased anxiety secondary to PTSD.^10^

Lastly, it was found that doses of 5 mg/kg/d prevented occurrence of graft‐*vs*‐host disease in a phase II clinical trial (*n* = 48) and 5–10 mg/kg/d doses have been shown in a case report to remove withdrawal symptoms from a patient with cannabis dependency.^29^, ^38^

Within studies that compared CBD against a placebo or control (*n* = 17 publications), only 1 compared CBD against an active control (and a greater clinical improvement and side effect profile was observed with CBD against amisulpride), 8 compared CBD against a placebo (monotherapy), and 8 studies compared CBD as an add‐on therapy (adjunctive to antipsychotic medication, antiepileptic medication, anti‐Parkinson medication or pain medication) against placebo. Analysis of these data revealed that a greater proportion of studies reported a beneficial effect of CBD in the add‐on therapy group compared to the monotherapy group (*n* = 6 and *n* = 2 respectively). However, higher doses were used overall within the add‐on therapy group compared to the monotherapy group (average 11 and 6 mg/kg/d, respectively) and, due to such a small data set and heterogeneity of studies, we did not perform any further analysis.

## 4 DISCUSSION

To our knowledge, this is the first study to compile and compare all publications in which CBD was administered to clinical populations. The aim of this systematic review was to better understand the range of doses of CBD used in clinical studies. In total, 13 medical contexts were included in this review amongst 35 studies including clinical trials and case reports. A positive effect of CBD was reported in 66% of studies, covering disorders including schizophrenia, SAD, epilepsy, cannabis dependency and graft‐*vs*‐host disease, with doses ranging between <1 and 50 mg/kg/d (i.e. <62–3100 mg/d for an adult). Although we acknowledge that these results mix widely heterogeneous studies, it appears well founded to highlight the differences in average dosing for positive effect studies against those without positive effects, which is confirmed when analysing studies per medical context within each study design format. This suggests that CBD potentially displays a wide therapeutic range, and variable minimum doses are required for effect depending on primary outcomes assessed and the population group. However, it is vital to note that no conclusions can be drawn on the efficacy of CBD as larger phase III and conclusive efficacy trials have not been conducted, with exception of epilepsy. A number of phase III clinical trials are registered on clinicaltrials.gov, which should provide more evidence in the coming years in the contexts of pain, anxiety, Crohn's disease, bipolar disorder, Fragile X syndrome, epilepsy and more.

CBD is increasingly popular, both as a food and health supplement and as a licensed medicine. Within this review, 51% of studies have been published in the last 5 years (since 2013); however, the included articles span over decades, with prominent publications first appearing in the 1980s and early 1990s.^24^, ^40^ Despite its long history of sole administration to patients, there is surprisingly little published about the pharmacokinetic properties of CBD, particularly its bioavailability, making it difficult to estimate true effective doses.^8^ Historically, there is a striking lack of dose‐ranging studies and, looking forward, there are no registered trials on clinicaltrials.gov including specific dose‐ranging investigations in their study design. Ideally, this review would have compared plasma concentrations of CBD in order to more accurately estimate therapeutic concentrations, but, due to the lack of reporting, this was not possible.

Different effective plasma concentrations of CBD may be required for achieving different endpoints across clinical populations, which is a recognised trait in a number of other drugs and diseases. For example, aspirin (acetylsalicylic acid) is used at low doses for antiplatelet therapy, and at higher doses as an analgesic agent.^44^, ^45^ With CBD, lower doses may be effective in anxiety relief, while higher doses may be required for effective reduction in epileptic seizures. In studies where there are good rationales for CBD use (e.g. Crohn's disease and chronic pain^46^, ^47^), neutral results may be secondary to subtherapeutic dosing, and dose‐escalation trials with embedded pharmacokinetic studies are the next logical step.^15^, ^22^ Studies in this review using higher doses concluded that CBD was generally well‐tolerated with the most frequent side effects including drowsiness, nausea, somnolence, fatigue and vomiting.

Among the clinical trial records retrieved from clinicaltrials.gov, only 60% of completed trials had results uploaded and available. This may represent a significant publication bias and is suggestive of disregard for the priority of publication of negative results, which is a well‐recognised problem.^48^ Unfortunately, this may potentially skew the findings presented in this review and so should be interpreted with caution and is acknowledged as a limitation. We also acknowledge that despite all routes of administration being oral, there may be further bias introduced between studies as one dose cannot be directly compared to another due to lack of standardisation of formulations and pharmacokinetic activity, including differences in bioavailability between an oral spray and an oral capsule.

Future studies should also consider the safety of drug interactions with CBD. CBD is a known inhibitor of the cytochrome P450 (CYP) system^49^ and can therefore increase plasma concentrations of medicines already in use, in particular antiepileptic drugs. Indeed, this has been reported in a number of publications investigating concomitant use of CBD and antiepileptic drugs.^50^ Similarly, CYP inhibitors are predicted to increase CBD plasma concentrations which should be equally monitored. Where possible, further well designed trials with CBD may disentangle whether CBD offers unique therapeutic potential in addition to benefits seen when used as an add‐on treatment.

## 5 CONCLUSION

Although larger confirmatory and efficacy clinical trials examining dosing in more detail for each medical context is required, this review summarises that CBD appears to offer a wide‐range of activity between 1 and 50 mg/kg/d, and there was a tendency of studies with positive outcomes to have used higher doses of CBD. We recommend pharmacokinetic dosing schedules in subsequent trials to consider this range along with safety data and individual patient requirements. Finally, we implore all completed trial results to be made readily available so the research community can progress and learn from equally important positive and negative outcomes for the ultimate benefit of patients.

## COMPETING INTERESTS

A.S.Y. and S.E.O. are paid consultants for Artelo Biosciences and the UK Centre for Medicinal Cannabis. All other authors declare no competing interests.

## CONTRIBUTORS

S.E.O. and S.A.M.: substantial contributions to the conception or design of the work. S.M.: writing of the manuscript. S.A.M., Z.D.B. and N.L.S.: database searching and data extraction. All authors: analysis and interpretation of data for the work; drafting the work or revising it critically for important intellectual content; final approval of the version to be published; agreement to be accountable for all aspects of the work in ensuring that questions related to the accuracy or integrity of any part of the work are appropriately investigated and resolved.
